# A Dictionary of Dental Science

**Published:** 1898-12

**Authors:** 


### Bibliographical.

A DICTIONARY OF DENTAL SCIENCE, and such words and phrases of the collateral sciences as pertain to the art and practice of dentistry. By Chapin A. Harris, M. D., D. D. S. Sixth edition. Carefully revised and enlarged by Ferdinand J. S. Gorgas, M. D., D. D. S. Philadelphia: P. Blakiston's Son & Co., 1012 Walnut Street, 1889. Price, cloth, $5.00; leather, $6.00. 662 pages.

This greatly improved work has the unique relation to our literature of never having had anything whatever in the shape of a rival. It never can be in better hands than that of Professor Gorgas, whose editorship of Harris' two works has been conscientiously and ably performed. Every dental student ought to own the dictionary. It is an absolute necessity to one who wants clear interpretation of the phrases of dental science. It does for dentistry precisely what an English dictionary does for our language. The present edition has been weeded of obsolete words, and has had many important additions made relating to the micro-organisms of the mouth, electricity, etc.—*Dominion Dental Journal.*

